# *Tinospora sinensis* (Lour.) Merr alkaloid rich extract induces colon cancer cell death via ROS mediated, mTOR dependent apoptosis pathway: “an in-vitro study”

**DOI:** 10.1186/s12906-023-03849-5

**Published:** 2023-02-03

**Authors:** Sreelakshmi Badavenkatappa gari, Vinod K. Nelson, Ramalingam Peraman

**Affiliations:** 1 Faculty of Pharmaceutical Sciences, Jawaharlal Nehru Technological University Anantapur (JNTUA), Anantapur, Andhra Pradesh 515 002 India; 2 Raghavendra Institute of Pharmaceutical Education and Research (RIPER) –Autonomous, Anantapur, Andhra Pradesh 515721 India; 3 Department of Pharmaceutical Analysis, National Institute of Pharmaceutical Education and Research (NIPER), Hajipur, 844102 India

**Keywords:** Colon cancer, *T. sinensis*, N-hexane extract of *Tinospora sinensis* root, ROS, Cell cycle, mTOR, Apoptosis

## Abstract

**Background:**

Colorectal cancer (CRC) is the second most mortality rate causing disease after lung cancer. Though there is a significant improvement in the treatment schedule offered to CRC. However, there is no notable decrease in terms of cases as well as death rate. Hence, there is an urgent need to discover novel cancer therapeutics to treat CRC. Since ancient times, the use of phytochemicals has drawn huge attention as chemo-preventive and chemotherapeutic agents. Earlier studies on *Tinospora sinensis* (TS) revealed the cytotoxic effect on human colorectal carcinoma (HCT-116) cells, yet the mechanism is to be uncovered. Therefore, the present study was designed to study the cell death mechanism of TS in HCT-116 cells.

**Method:**

Different extracts such as n-hexane, ethyl acetate, and ethanol extracts from the root part of TS were prepared using a cold maceration process. The extracts were screened against cancer cell lines by methyl thiazoldiphenyltetrazolium bromide (MTT) assay. From the result, the most active extract was subjected to gas chromatography-mass spectrometry (GC-MS) and Fourier-Transform infrared spectroscopy (FTIR) analyses to identify the major constituents. Finally, the mechanism of cytotoxicity to cancer cells for the most active extract was evaluated using various experiments such as cell cycle analysis, Annexin-V assay, and Western blot.

**Results:**

The results from the MTT assay indicated that the n-hexane extract of TS inhibits the growth of HCT-116 cells more effectively than other cancer cells like Henrietta Lacks cervical cancer cells (Hela), and Michigan cancer foundation-breast cancer (MCF-7). The GC-MS and FT-IR analyses revealed the presence of alkaloids in the n-hexane extract and were responsible for the apoptosis activity in HCT-cells via reactive oxygen species (ROS) generation, and phosphoinositide 3-kinase (PI3K)/ protein Kinase B (Akt)/ mammalian target of rapamycin (mTOR) down-regulation.

**Conclusion:**

This study concludes that this finding is unique of its kind, and for the first time. The anticancer effect of TS root is specific to colon cancer cells (HCT-116). This distinctive finding helps the researchers to investigate further, and to identify a novel source for anti-colon cancer drug candidates in near future.

**Supplementary Information:**

The online version contains supplementary material available at 10.1186/s12906-023-03849-5.

## Background

Cancer disease is a growing health concern, becoming a huge burden to mankind, and it affects millions of people every year. Worldwide, colorectal cancer (CRC) is the third most frequently occurring cancer as well as the second most death-causing disease [1, 2]. According to recent statistics, about 1.9 million people were diagnosed with CRC in the year 2020. In addition, one in 10 cancer deaths are due to CRC [2]. Moreover, CRC is more gender specific, where the male population is more affected than the female [3].

There are several risk factors such as genetic, age, epigenetic changes, and hereditary factors that play a prominent role in the development of CRC [4, 5]. There are other factors like increased sedentary lifestyle, consumption of processed meat, alcohol, cigarette smoking, obesity, and stress also contribute much to the progress of CRC [4, 6].

The current treatment approaches available for CRC include radiation therapy, surgery, endoscopy, and supplementation of chemotherapeutic agents. However, these treatments are too costly to reach low-income patients. Besides, they were also associated with severe toxicity. Hence, there is an ultimate necessity for newer drug treatments for colon cancer and is continuing interest among researchers.

From ancient times, human beings predominately depended on plants and plant-derived products to treat various diseases [7–10]. They played an important role in the discovery of bioactive compounds for several diseases, especially cancer. Several reports suggested that plant extracts or plant-derived secondary metabolites always serve as a promising source for drug discovery for many diseases including cancer. Their uniqueness is because of their negligible toxicity, fewer side effects, biocompatibility, and biodegradability [11, 12].

Among the phytoconstituents, the alkaloids and polyphenol compounds are well-regarded metabolites that exhibit pronounced inhibition of cell growth. It was known that the anticancer effect of these compounds is not only due to antioxidant properties but also due to the pro-oxidant effect [11, 13–15].

Several studies revealed that colon cancer cells require high reactive oxygen species (ROS) that help to survive with their high metabolic rate [16]. However, an increased level of ROS higher than the threshold limit thrusts the cancer cells to death. Hence, the plant extracts or the phytochemicals that induce ROS, can contribute as an anticancer drug by initiating cell death by modulating multiple signaling pathways in cancer cells [17]. In addition, reports also suggested that ROS induces apoptotic cell death in colon cancer cells via down-regulating a variety of signaling molecules such as PI3K/AKT/mTOR and up-regulating caspase proteins (Caspase 3 and 9) [18].

In our current study, we have chosen the plant, *Tinospora sinensis* (Family: Menispermaceae) as a natural source of anticancer agents against colon cancer (HCT-116) cells. Mostly, *Tinospora* species grow in tropical and subtropical areas such as Asia, Africa, and Australia. This plant was traditionally used for treating fever, cold, pharyngitis, oral ulcer, digestive disorders, rheumatoid arthritis, and immune deficiency [19, 20]. Moreover, *Tinospora* species were widely used as one of the ingredients in various traditional formulations to treat different kinds of diseases [21].

However, the anticancer effect of *Tinospora sinensis* (TS) was not well studied. Hence the present study aimed to investigate the anticancer effect of TS for its root extract against HCT-116 cells and to evaluate the mechanism of cell killing via multiple experiments such as cells cycle analysis, Annexin V assay, and Western blot experiments.

## Materials and methods

### Chemicals, reagents, and cell lines

HCT-116, MCF7, and Hela cells obtained from the National Center for cell science (NCCS, Pune), Dulbecco’s modified eagle medium (DMEM- High Glucose), fetal bovine serum (FBS), MTT reagent (5 mg/ml), phosphate-buffered saline (PBS) procured from Hi-Media. India. Camptothecin, dimethyl sulfoxide (DMSO) supplied by Sigma-Aldrich. Propidium iodide, acridine orange, 2,7-dichlorofluorescein (DCFDA acquired from Thermo Fischer, USA. Primary antibodies like caspases (3, 7, 9), phospo Akt, Akt, phospho ERK, ERK, mTOR, Bcl-2, β-actin, and anti-mouse secondary antibodies were acquired from cell signaling technology (CST).

### Collection of plant material

Fresh roots of TS were collected from Anantapuramu, Andhra Pradesh, India. Then, it was authenticated by Dr. B Ravi Prasad Rao, Professor, Biodiversity Conservation Division, Sri Krishnadevaraya University (SKU), Anantapur, Andhra Pradesh, India. Finally, the specimen in the form of herbarium with a voucher number *55585* was deposited at the department of botany, Sri Krishnadevaraya University (SKU), Anantapuramu. India.

### Preparation of plant extracts

The collected root material was dried under shade and made into fine powder. Weighed 500 g of the powdered plant material and was subjected to successive solvent extraction using different polarity solvents such as n-hexane, ethyl acetate, and ethanol by the maceration method. Subsequently, each extracted content was filtered using Whatman No. 1 filter paper and evaporated using a rotary vacuum evaporator, and finally, all the extracts were stored at 4 °C till further use. The same procedure was replicated three times for each extract. In the end, the respective extract was combined and stored properly until usage. One hundred milligrams of each solvent extract was accurately weighed and dissolved in 10 ml of DMSO (dimethyl sulphoxide). These DMSO stocks were stored at − 20 °C until use. In all the cell experiments, the final concentration of DMSO was maintained at less than 0.25% (a non-toxic concentration).

### Phytochemical analysis of extracts

After extraction, about 1 mg of each extract of n-hexane, ethyl acetate, and ethanol solvents, was taken for analyzed the presence of various primary and secondary metabolites like carbohydrates, proteins, glycosides, alkaloids, flavonoids, terpenoids, tannins, saponins and polyphenols using various standard methods. The obtained results were tabulated and shown in Table 1.

### Qualitative analysis of secondary metabolites

#### Alkaloids

Approximately, 1 mg of each extract was taken into a beaker containing 5 ml of diluted HCl separately. Each beaker was kept in the water bath for at least 5 min and then filtered. The filtrate of each extract was divided into two portions. To one portion, 3–5 drops of Dragendorff’s reagent were added, and the appearance of an orange-red precipitate indicated the presence of alkaloid compounds. Similarly, to other portions of the filtrate, Mayer’s reagent was added; observation of buff-color precipitate indicated the presence of alkaloids [22, 23].

#### Amino acids

Approximately 1 mg of each solvent extract was taken in a separate test tube and mixed with 1 ml distilled water each. Then, a few drops of Ninhydrin reagent were added to it and kept under-heating. The generation of purple color indicated the presence of amino acids in the extract [24].

#### Carbohydrates

One milligram of each extract was taken separately in a test tube, and was mixed with 2 ml of distilled water and vortexed. To this mixture, 1 ml of concentrated sulphuric acid was added gently by the walls of the test tube. The observation of a purple color precipitate in the solution indicated the presence of carbohydrates in the extract [23].

#### Diterpenoids

One milligram of each extract was taken separately and added 1 ml chloroform solvent and mixed it thoroughly. To this mixture, 1 ml of concentrated sulphuric acid was added by the walls of the test tube. The appearance of color in the solution indicated the existence of terpenoids in the extract [25].

#### Flavonoids

One milligram of each solvent extract was taken separately and dissolved in 2 ml of distilled water in a separate test tube and vortexed. To this solution, 1 ml of concentrated sulphuric acid was added gently. The development of yellow color in the solution indicated the presence of flavonoids [25].

#### Glycosides

One milligram of each extract was taken separately and added to the mixture that contains an equal quantity of dilute HCl, sodium nitroprusside in pyridine solution, and sodium hydroxide. The formation of pink to blood-red color inferred the presence of glycosides [23].

#### Phenols

For each 1 mg of the respective extract solution, 3–5 drops of neutral 5% ferric chloride were added. The appearance of dark green color indicated a positive test for phenolic compounds [26].

#### Saponins

To the 1 mg of each extract added 5 ml of distilled water and kept under boiling for 5 min. After boiling, the filtrate of each extract was collected individually and added with 3 ml distilled water to each test tube. Then, the content was shaken vigorously for at least 5 min. The appearance of the frothing even after warming indicates the presence of saponins [23, 27].

#### Steroids

To 1 mg of each solvent extract in the separate test tube, 5 ml chloroform was added and later the same volume of concentrated sulphuric acid was added to it by the side of the test tube walls gently. The immediate development of red color in the chloroform layer and yellow with green fluorescence indicated the presence of steroids in the extract [23].

#### Tannins

About 1 mg extract of each solvent was added to each test tube containing 2 ml distilled water and vortexed. To this mixture 3–5 drops of 0.1%, ferric chloride was added. The appearance of a blue-black precipitate indicated the presence of tannins in the extract [25].

### GC-MS analysis

The n-hexane extract was subjected to GC-MS analysis (GC-MS SHIMADZU GC-2010) using a short (< 5 m) sin column with a constant mass flow rate of carrier gas (helium). Initially, the oven temperature was maintained at 70 °C for 2.0 minutes, and the temperature was gradually increased up to 300 °C at 10.0/35.0 minutes and 4.0 μl of the sample was injected for analysis. The flow rate of helium gas was set to 1 .5 ml/min. The sample injector temperature was maintained at 260 °C and the split ratio was 20 throughout the experiment. The ionization was done with 70 eV. Finally, the mass spectra were recorded for the mass range of 40–1000 *m/z* for about 35 minutes. The chromatogram and mass-to-charge ratio spectra were used in the identification of compounds [28].

### MTT assay/cytotoxic assay

The day before the experiment, all the cancer cells such as HeLa, HCT-116, and MCF-7 cells were plated in the 96-well plate and allowed to grow to reach 70% confluence. Subsequently, the cells were treated with DMSO (vehicle control), with different concentrations of each extract (n-hexane, ethyl acetate, and ethanol) like 50, 150, 250, 350, and 450 μg/ml. Then incubate each extract-treated cell for about 24 h. After the stipulated time, the MTT reagent of 0.5 mg/ml concentration was added to each well and kept for 3 h. After 3 h MTT reagent was removed and added 300ul of DMSO and kept under continuous shaking for 20 min. Finally, the absorbance value was obtained using an ELISA reader at 570 nm. Afterward, the % inhibition for each concentration for each extract against test cell lines was obtained using Graph pad prism version 5.0 and acquired the IC50 value of each extract on each cancer cell [10].

### Cell cycle analysis

Based on the MTT assay result, the HCT-116 cells were seeded in 12 well plates at a density of 1 × l0^5^ cells/well and subjected to starvation for 6 hours before treatment. After a starvation period, the cells were treated with DMSO control and n-hexane extract (at IC-50 concentration) for 24 hours. Then cells were collected, washed with PBS, and fixed in 70% ethanol at − 20 °C. Fixed cells were stained with PI buffer of about 350 μL at 37 °C for 30 min in a dark place, and then finally analyzed using FACS [29].

### Assessment of cell morphology: Acridine orange-ethidium bromide staining

HCT-116 cells were seeded in 24 well plates at a density of 5 × 10^4^ cells/well and incubated for 24 hours with vehicle control (DMSO) and n-hexane extract at IC-50 concentration. After 24 hours, added the acridine orange-ethidium bromide of 100 μg/ml was to each incubated cell to distinguish the live, apoptotic, and necrotic cells in treated and untreated cells using a fluorescence microscope with excitation (488 nm) and emission (550 nm) at 200X magnification. Finally, results were obtained and plotted [30].

### DAPI staining

HCT-116 cells were grown in a 24-well plate at a seeding density of 5 × 10^4^ cells/well and treated with DMSO control, n-hexane extract (at IC-50 concentration), and a standard (Camptothecin 15 μM). After 24 hours of incubation time, the media was removed and washed with PBS twice. A volume of 200 μL of 0.2% triton X was added and incubated for 10 minutes. Excess, triton X was removed and stained with 1 μM of DAPI and observed under the fluorescence microscope with an excitation wavelength of 359 nm and emission wavelength at 460 nm using a DAPI filter at 200 X magnification. The results were obtained and plotted [31].

### CASPASE 3 expression

HCT-116 cells were plated in a 6-well plate at a density of 3 × 10^5^ cells and treated the cells with DMSO, n-hexane extract (at IC-50 concentration), and standard compound (Camptothecin 15 μM) for about 24 h. At the end of the treatment, the medium was removed and washed with PBS. Afterward, the cells were trypsinized with a trypsin-EDTA solution, and then cells were collected in fresh separate 1.5 ml centrifuge tubes. To these cell pellets, 1 ml of chilled 70% ethanol was added and fixed the cells for about 30 min on the ice. To the ethanol-fixed cells, recommended concentration of FITC caspase 3 antibodies was added and incubated for about 30 min at room temperature in a dark place. After the incubation time, the cells were washed with PBS, 0.1% sodium azide was added, mixed thoroughly, and analyzed using FACS [32].

### Analysis of mitochondrial membrane potential (MMP) by JC 1 staining

HCT-116 cells were plated in a 12 well-plate and allowed to grow to reach 70% confluence and were treated with DMSO control and n-hexane extract (50 μg/ml) and a standard compound (15 μM). After a specific incubation time, a JC1 stain of 3 μM concentration was added to the wells and incubated for 45 minutes. Then, the cells were trypsinized and centrifuged for 5 min at 1610×g. The pellet was collected and suspended in PBS. Finally, the data was acquired using FACS and plotted [33].

### Annexin V binding assay

The day before the experiment, HCT-116 cells were seeded in a 12-well plate at a density of 1.5 × 10^5^/well. The next day, the media was changed and treated with vehicle control (DMSO), n-hexane extract (50 μg/ml), and standard camptothecin (15 μM) for 24 h. After the incubation time, the cells were trypsinized and centrifuged at 2516×g for 3 minutes. The cell pellet was collected and washed with PBS buffer. Then, processed for apoptosis with Alexa fluor™ 488 Annexin V/Dead cell apoptosis kit according to the manufacturer’s protocol [33].

### Protein extraction and western blotting

HCT-116 cells were seeded uniformly in a 60 mm dish and treated with DMSO, n-hexane extract, and standard compound for 24 h. After the incubation time, the cells were homogenized using RIPA buffer (50 mM Tris, pH 8.0, 150 mM NaCl, 1% NP-40, 0.5% sodium deoxycholate, 0.1% SDS, and a complete protease inhibitor cocktail). The lysed samples were centrifuged at 14489×g for 10 min, and the supernatant was collected separately. Finally, the protein was measured by the BCA method and stored at -80 °C till use. The protein sample of the equal amount was loaded and separated using sodium dodecyl sulfate-polyacrylamide gel electrophoresis (SDS-PAGE), and then the immunoblotting was done. The primary antibodies such as caspases (7, 9) mTOR, p-Akt, p-ERK, and Bcl-2 were used at 1:1000 dilutions and the β-actin antibody was used at 1:5000 dilutions. At the end of the experiment, the data was obtained and represented in fold change in comparison with the control as well as standard [10, 34].

### Statistical analysis

The results from all series of experiments were performed in triplicates (*n* = 3) and expressed as mean ± standard error mean using multiple comparisons of significant analysis of variance (ANOVA) followed by Dunnett’s test as post parametric test using a computer-based fitting program (Prism graph pad version 5.0). A *p*-value of less than 0.05 is considered statistically significant (compared with the control). Moreover, all methods were carried out according to the relevant guidelines and regulations.

## Results

### Phytochemical analysis of extracts

The crude extracts obtained from *Tinospora sinensis* root were resinous, and the respective percentage yield of n-hexane (NHTR), ethyl acetate (EATR), and ethanol (ELTR) extracts are 6.2, 8.9 and 8.4%. After extraction, each extract was analyzed for the presence of various classes of phytoconstituents. The preliminary phytochemical analysis data revealed the presence of various phytoconstituents in different extracts as shown in Table 1. From the data, it was inferred that an abundant amount of alkaloids was present, more specifically the NHTR enriched with higher alkaloidal contents when compared to other extracts. The further identification of alkaloids in the NHTR was performed using analytical experiments such as GCMS and FT-IR analyses.

**Table 1:** Qualitative analysis of phytochemicals in different extracts of *T.sinesis* roots shows the presence of various classes of secondary metabolites. (+ indicates presence, ++ moderately present, +++ highly abundant)

Preliminary qualitative Phytochemical analysis			
	NHRT	EART	ELRT
**Alkaloids**	**+++**	**+**	**+**
**Steroids**	**+**	**–**	**–**
**Glycosides**	**+**	**+**	**+**
**Amino acids**	**–**	**+**	**+**
**Carbohydrates**	**++**	**+**	**+**
**Flavonoids**	**–**	**–**	**–**
**Tannins**	**–**	**–**	**–**
**Saponins**	**+**	**+**	**+++**
**Diterpenes**	**+**	**+**	**++**

### NHTR shows a significant anticancer effect on colon cancer cell HCT-116

**Fig. 1:**
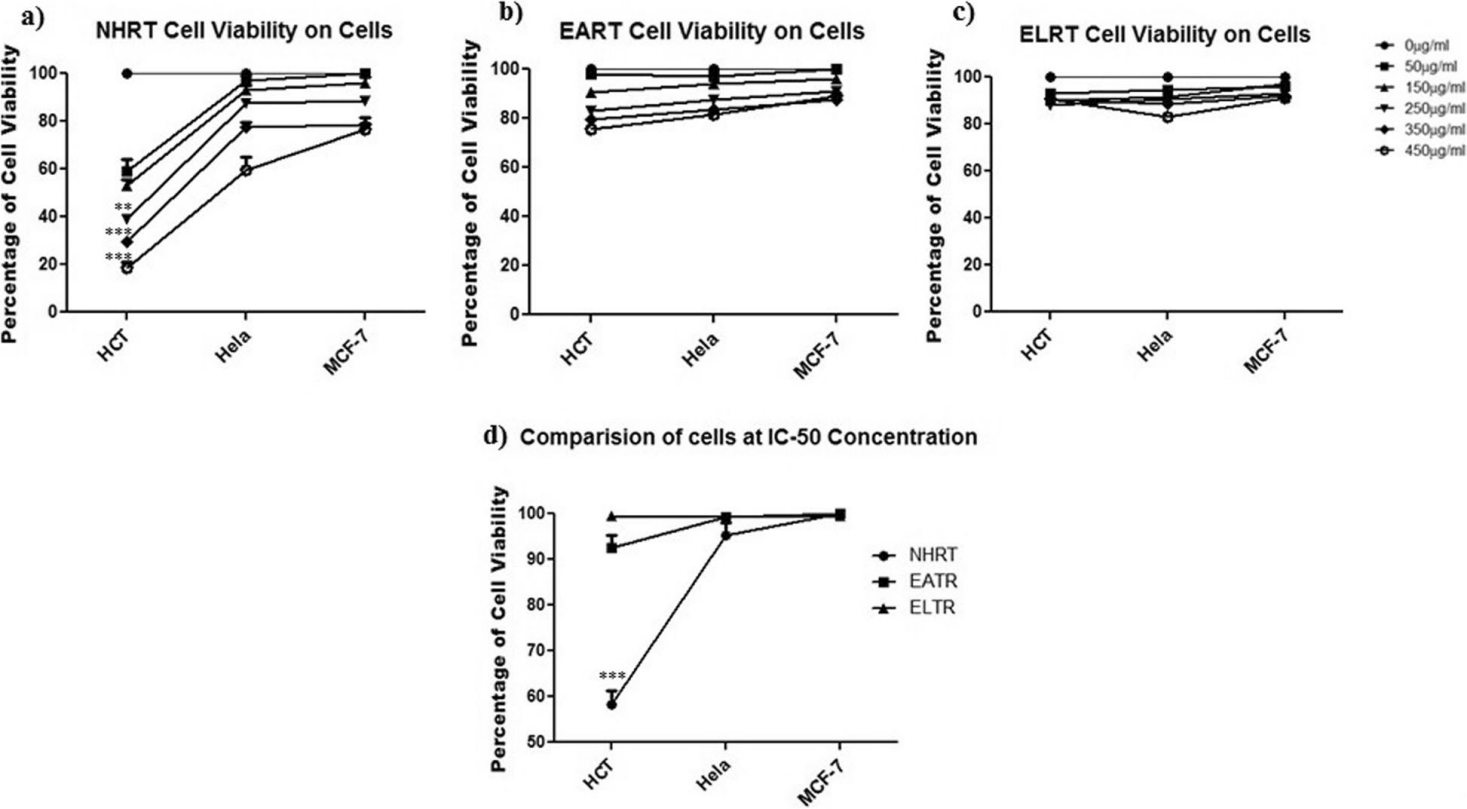
The data shows NHRT exhibit cytotoxic activity on colon cancer cells HCT-116 as compared to other cancer cells HeLa and MCF-7 cells (graph **a**, **b, c** & **d**). All the data and expressed as Mean ± SEM. *p* value (*p* < 0.05) is considered as statistical significance. (**p < 0.05, *** *p* < 0.01) when compared to control group

Each of the extracts was treated with cancer cell lines like HCT-116, MCF-7, and Hela cells at different concentrations such as 0, 50, 150, 250, 350, and 450 μg/ml. DMSO was used as a control. The MTT assay was carried out to identify the % inhibition on the growth of cancer cells. The MTT assay result revealed the NHTR extract as the most active in comparison with the other extracts (EATR and ELTR) (Fig. 1). The IC-50 values (Shown in Table 2) disclosed that NHTR was more specifically targeting the colon cancer cells (HCT-116 cells), but was less active on Hela and MCF-7 cells (Fig. 1). The IC-50 values of NHTR on HCT-116, Hela, and MCF-7 cells were 42.8 ± 680, 208.0 ± 16.64, and 541.2 ± 2.005, respectively. This assay revealed that the abundant phytochemicals present in the NHRT plays role in producing a cytotoxic effect in HCT-116 cells. Hence, further, the following experiments were carried out on HCT-116 cells at IC-50 concentration.

**Table 2:** Shows the IC-50 value of different extract for different cancer cell. It also indicate that NHRT is highly specific to the colon cancer cells (HCT-116)

S. NO	Extracts	IC_**50**_ value		
		HeLa	HCT-116	MCF-7
1	**NHTR**	208.0 ± 16.64	42.48 ± 1.680^**^	541.2 ± 2.005
2	**EATR**	226.7 ± 12.54	356.45 ± 4.160^**^	521.6 ± 2.135^*^
3	**ELTR**	385.2 ± 2.005	409.7 ± 6.755	556.0 ± 9.330

^**^Highly Significant, ^*^Significant

### NHTR induces G0/G1 phase arrest in HCT-116 cells

**Fig. 2:**
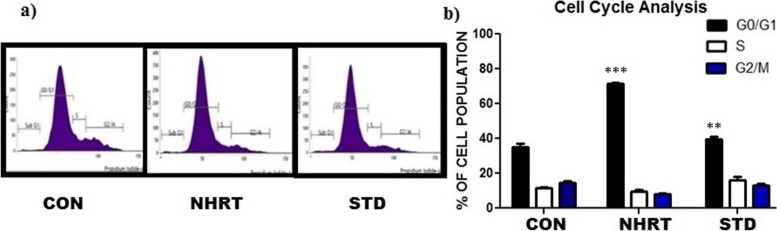
Panel **a)** Shows NHRT at IC-50 concentration induce cell death in HCT-116 cells via cell cycle arrest at G0/G1 phase as compared with control (DMSO) and Camptothecin (15 μM). Panel **b)** shows the percentage of cell population in each phase of the cell division. NHRT increase cell population in G0/G1 as compared with other phase as well as other groups. All the data were expressed as Mean ± SEM *p* value (*p* < 0.05) is considered as statistical significance. (**p < 0.05, *** *p* < 0.01) when compared to control group

Several reports stated that plant constituents induce apoptotic cell death in cancer cells either directly or by triggering cell cycle arrest [29, 35, 36]. From the MTT assay, we identified the NHTR as a potent extract for its cytotoxicity, further it also induces cancer-specific cell death in the HCT-116 cells. To identify the mechanism of NHTR in HCT-116 cells, a cell cycle analysis experiment was conducted. FACS analysis was done using control (DMSO), NHRT (50 μg/ml), and standard (Camptothecin;15 μM). The results revealed that NHRT induces significant cell death in HCT-116 cells via arresting the cell division at G0/G1 phase (Fig. 2). Moreover, there was a significant increase in the cell population at G0/G1 phase in NHRT treated group as compared with the camptothecin (15 μM) treated sample. This indicated that NHRT induces cell cycle arrest in HCT-116 cells.

### NHRT induces apoptosis in HCT-116 cells

**Fig. 3:**
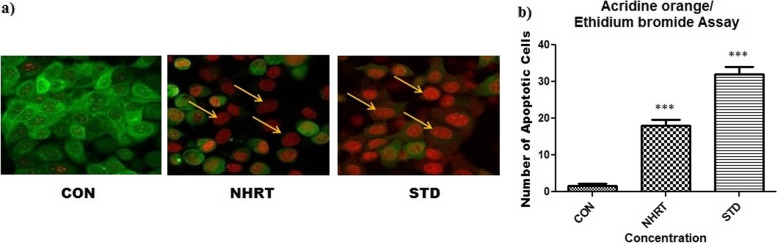
Panel **a)** showing live cells (green) and apoptotic cells (red), the arrow showing the apoptotic features such as plasma membrane blebbing, condensed nuclei and apoptotic body formation in the NHRT treated group and Camptothecin treated group. The Panel **b)** Number of apoptotic cells counted in each group in a particular field. All the data were expressed as Mean ± SEM p value (*p* < 0.05) is considered as statistical significance. *** *p* < 0.01 when compared control vs treated group

**Fig. 4:**
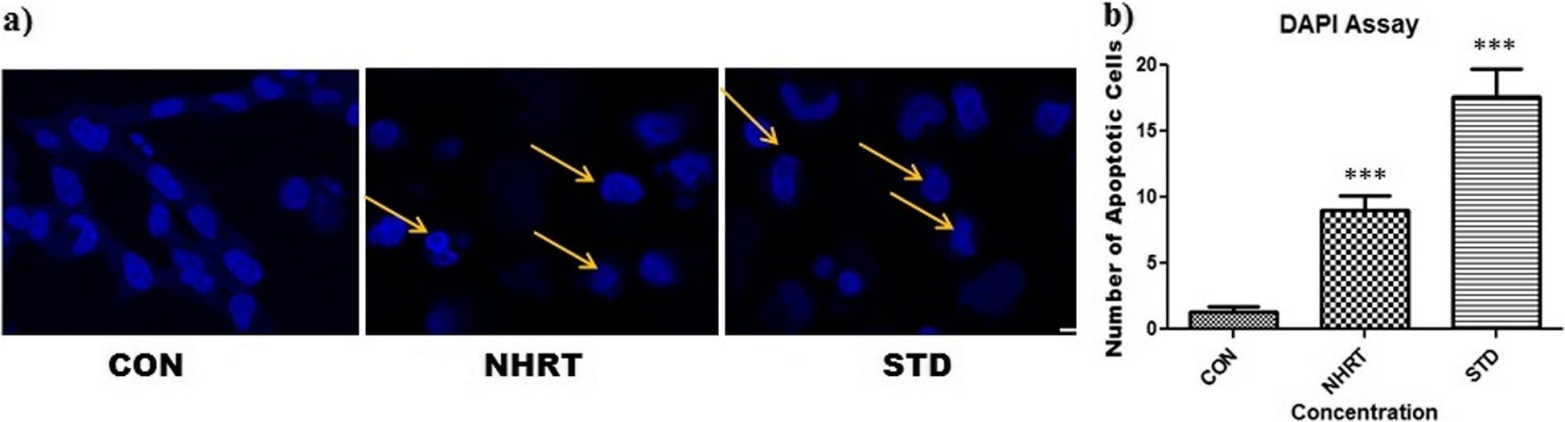
Panel **a)** Shows significant morphological change in the nucleus shape, membrane blebbing, horse shoe shape nuclei formation in the NHRT treated group and Camptothecin treated group when compared with control group. Panel **b)** Number of apoptotic cells counted in each group in a particular field. All the data were expressed as Mean ± SEM p value (*p* < 0.05) is considered as statistical significance. *** *p* < 0.01 when compared control vs treated group

**Fig. 5:**
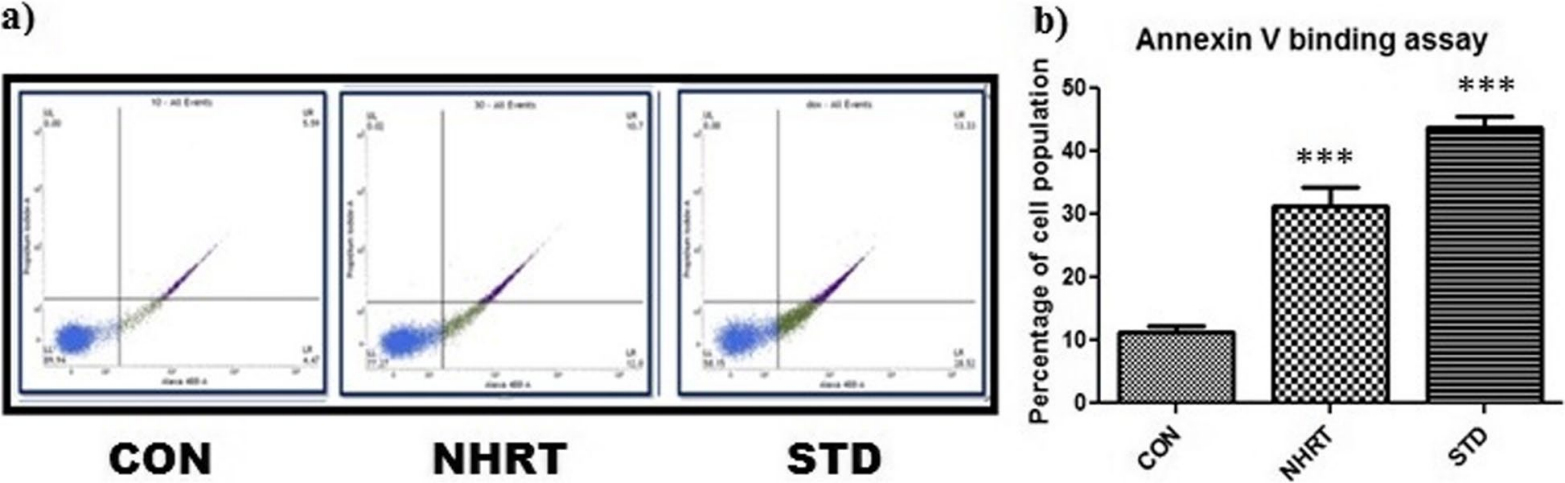
Panel **a)** Shows the increase in apoptotic population (early and late apoptotic) in NHRT treated as well as camptothecin treated groups. Panel **b)** clearly indicates the increase in percentage population of apoptotic cells in the treatment groups. All experiments were repeated at least three times to obtain the data. Finally the data were expressed as Mean ± SEM *** *p* < 0.01 control vs treatment

The previous experiment results concluded that the NHRT triggers cell death by inducing cell cycle arrest at G0/G1 phase. However, the mechanism of cell arrest was not explored. Therefore, AO/EB double staining experiment in HCT-116 cells was performed for NHRT using control, and standard. The results indicate that there was a significant decrease in the number of cells in the NHTR-treated group and camptothecin 15 μM (standard) group as compared to the control (DMSO) group. In addition, the images show distinct apoptotic features such as plasma membrane blebbing (late stage of apoptosis), condensed nuclei, and apoptotic body formation. Similar to the standard, the NHTR-treated group showed highly intense red color stained cells significantly in comparison to the control group (Fig. 3). This concluded that the extract induces cell death by apoptosis mechanism. Hence, for further confirmation, we also performed the DAPI staining of the cells. The results showed a notable change in the nucleus of the NHTR-treated groups similar to the standard group. The observed changes are membrane blebbing, formation of horseshoe shape nuclei, and fragmentation. This confirmed that NHRT triggers apoptosis in HCT-116 cells (Fig. 4). In the same way, Annexin V/PI staining of HCT-116 cells was performed for NHRT and standard groups. The results indicated a remarkable increase in the percentage of apoptotic (early and late) cells in the NHRT extract group (28.87%) and standard group (41.76%) as compared with the control group (10.06%) (Fig. 5).

**Fig. 6:**
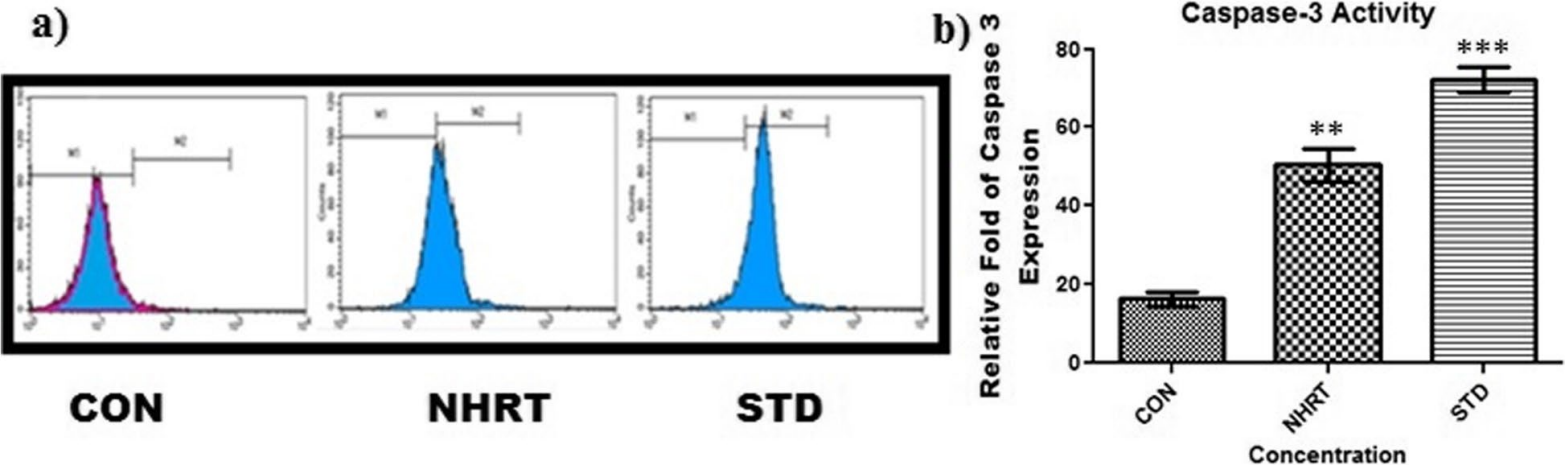
Panel **a)** Shows increase in Caspase 3 expression after the treatment of n-Hexane extract (NHRT) and standard compound (camptothecin). In above fig. M1 refers to Negative expression/region of caspase-3 and M2 refers to the Positive expression/region of caspase-3. Panel **b)** shows relative fold of caspase-3 expression in various groups. All the data were expressed as Mean ± SEM p value (*p* < 0.05) is considered as statistical significance. ** *p* < 0.05, *** *p* < 0.01 when compared control vs treated group

**Fig. 7:**
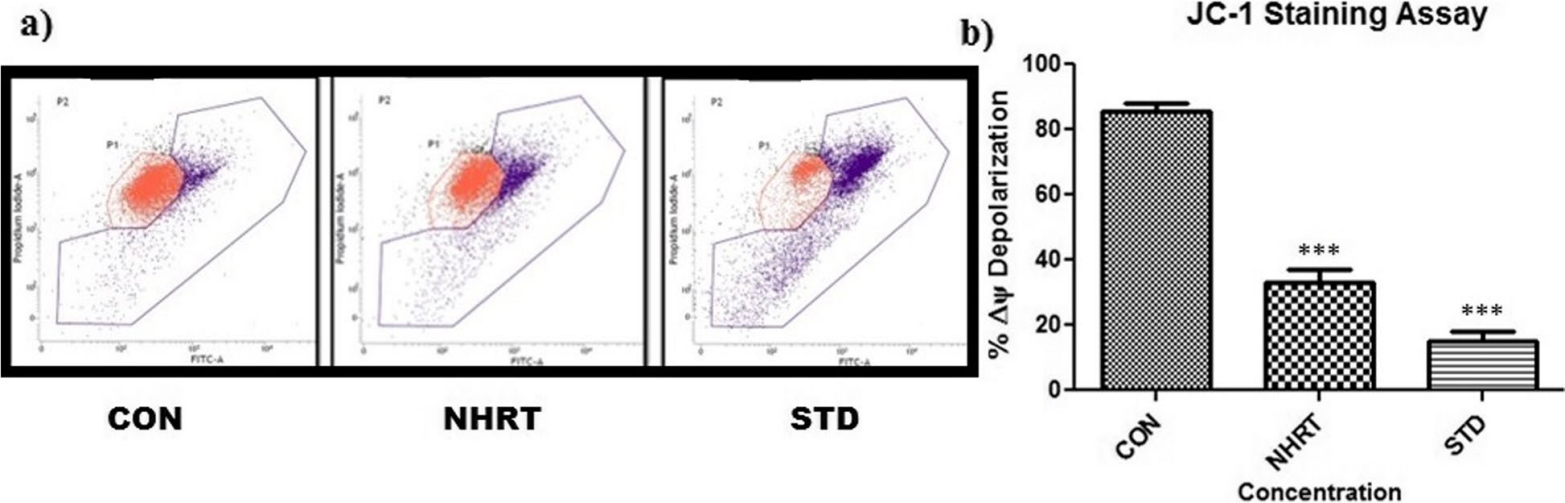
Panel **a)** FACS analysis shows change in the mitochondrial membrane potentials (MMP) in NHRT group and Standard in comparison with control group. Blue fluorescence indicates increase of JC1 stain which is an indicator of damage in the membrane of mitochondria and leads to loss its potential. Panel **b)** indicates quantification of MMP in different groups. All the data were expressed as Mean ± SEM p value (*p* < 0.05) is considered as statistical significance. *** *p* < 0.01 when compared control vs treated group

**Fig. 8:**
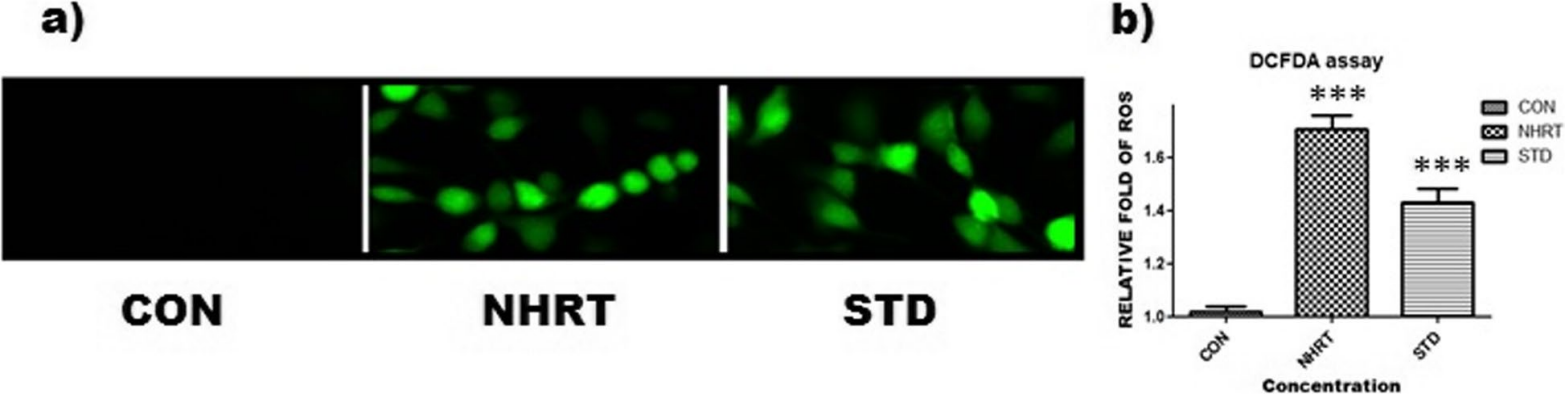
Panel **a**) Shows that NHRT and camptothecin treated group generate intracellular ROS in HCT − 116 in comparison with control group. Panel **b**) indicates the relative fold of ROS in different treated group. All the data were expressed as Mean ± SEM p value (*p* < 0.05) is considered as statistical significance. *** *p* < 0.01 when compared control vs treated group

Besides, NHRT also induces caspase-3 expression in the HCT-116 cells as compared with the control (Fig. 6). Generally, the M1 quadrant represents many living cells with no activation of caspase-3, whereas the M2 quadrant represents apoptotic cells with activated caspase-3. In the experimental result, there was a significant increase in the cell population in the M2 quadrant specifically in NHRT and standard-treated cells. On the other hand, there was no increase in cell population in the M2 quadrant in the control group (Fig. 6). This concluded that NHRT induces apoptosis in HCT-116 cells via caspase 3 activation. In continuation, we experimented to establish the relation of caspase 3 activations to mitochondrial dysfunction in apoptosis. Here, we found that NHRT and standard groups significantly reduce the mitochondrial membrane potential (MMP) as compared to the control group, where JC-1 acts as an indicator of MMP (Fig. 7). In this experiment, the NHRT suppressed the MMP, thus indicating the accumulation of the JC-1 monolayer (Blue fluorescence). These results confirmed that NHRT triggers apoptosis in HCT-116 cells through mitochondrial dysfunction. It was evident in the DCFDA experiment where NHRT induced upregulation of intracellular ROS and mitochondrial dysfunction in HCT-116 cells (Fig. 8).

### NHRT initiates mTOR-dependent apoptosis via PI3K/AKT pathway

**Fig. 9:**
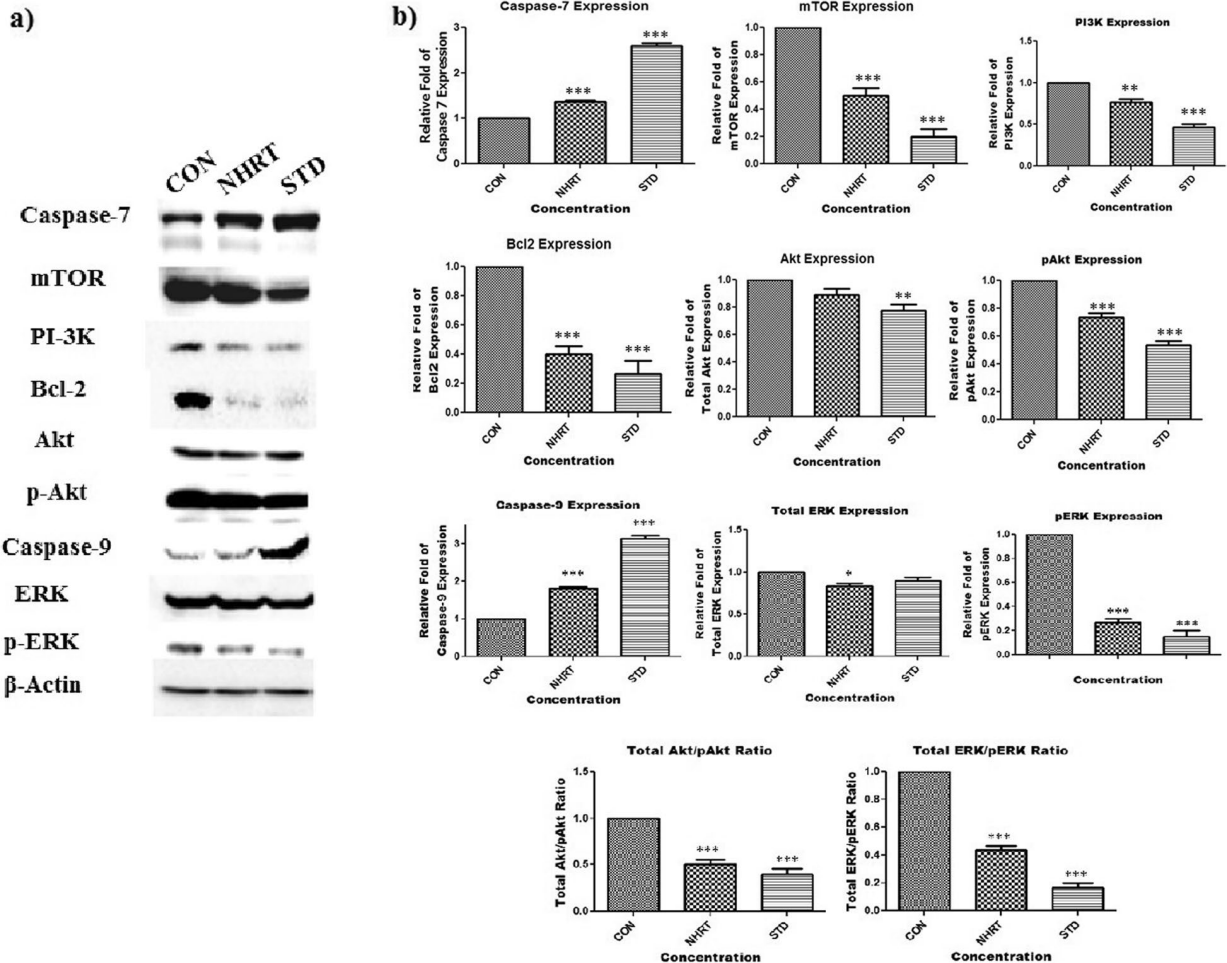
Panel **a)** Showing the immunoblot that represents the effect of indicated concentration of NHRT (IC-50 concentration) as well as camptothecin (15 μM) on various protein levels. β- Actin was used an internal loading control. Panel **b)** shows quantification of apoptotic markers. All the data were expressed as Mean ± SEM p value (*p* < 0.05) is considered as statistical significance. ** *p* < 0.05, *** *p* < 0.01 when compared control vs treated group

In colon cancer cells, the mammalian target of rapamycin (mTOR) is always in an active state and is mainly responsible for cell growth, survival, and metabolism. Moreover, mTOR is the major downstream target of the PI3K/AKT pathway. In a stress environment, the cell incorporates both the caspase family and mTOR signaling for regulating cellular function [37]. At this juncture, inhibition of mTOR and its associated signals along with activation of caspases such as 3, 7, and 9 shall lead to cell death. Earlier reports suggested that forced inhibition of mTOR by chemotherapeutics always results in the triggering of apoptosis via activation of caspase-3 function [37]. In our western blot experiments, we found that NHRT (50 μg/ml) and standard (camptothecin 15 μM) activate apoptotic cell death in HCT-116 cells via an extrinsic pathway. The results (Fig. 9 & Fig. S1) revealed that NHRT down-regulates phosphatidylinositol 3-kinase PI3K/Akt signals, and its downstream signal mTOR. The NHRT inhibited the anti-apoptotic protein Bcl-2, furthermore, it up-regulated both caspase 7 and caspase 9 significantly in comparison to the control group. From the literature, it is known that the Ras/PI3/Akt/mTOR pathway is coordinated with the Ras/Raf/Mitogen-activated protein kinase (MEK)/extracellular signal-regulated kinase (ERK) pathway for controlling cell proliferation. Hence, modulation of p-ERK/ERK along with the mTOR and its associated signals leads to apoptotic cell death in cancer cells. Similarly, our experiment results (Fig. 9 & Fig. S1) showed that NHRT inhibited the mTOR pathway in addition to the inhibition of p-ERK, and induced apoptosis [37].

### GC-MS analysis of n-hexane extract of *T. sinensis* root

**Fig. 10:**
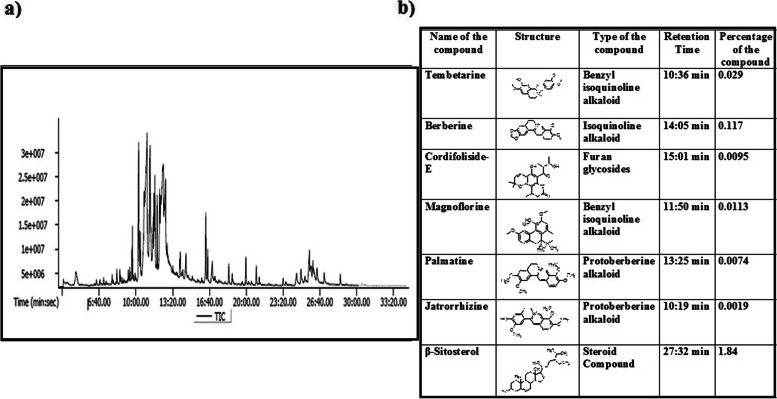
Panel **a)** Shows GC-MS chromatogram and there corresponding compounds with their retention times. Data clearly indicates the presence of alkaloids. Panel **b)** shows type of compound, Retention time, structure and Percentage

It is known that medicinal plant shows multiple biological functions due to presence of various characteristic phytochemicals. In our study the n-hexane extract also demonstrated a potential anticancer effect against colon cancer cells (HCT-116) specifically. Here, it is important to identify the actual compounds responsible for the observed anticancer activity. Hence, GC-MS analysis was conducted for NHRT. The results revealed the presence of several compounds in NHRT. A careful examination of peaks in the chromatogram, and their m/z value confirmed the presence of a few alkaloids and other compounds, namely tembetarine (*m/z* 344.4), berberine (*m/z* 336.4), magnoflorine (*m/z* 342.41), palmatine (*m/z* 352.4), Beta-sitosterol (m/z:414.7), cordifoliside E (m/z:372) and jatrorrhizine (m/z:338) (GC-MS chromatogram and retention time of respective compounds (Shown in Fig. 10a&b).

### FTIR analysis of n-hexane extract of *T. sinensis* root

**Fig. 11:**
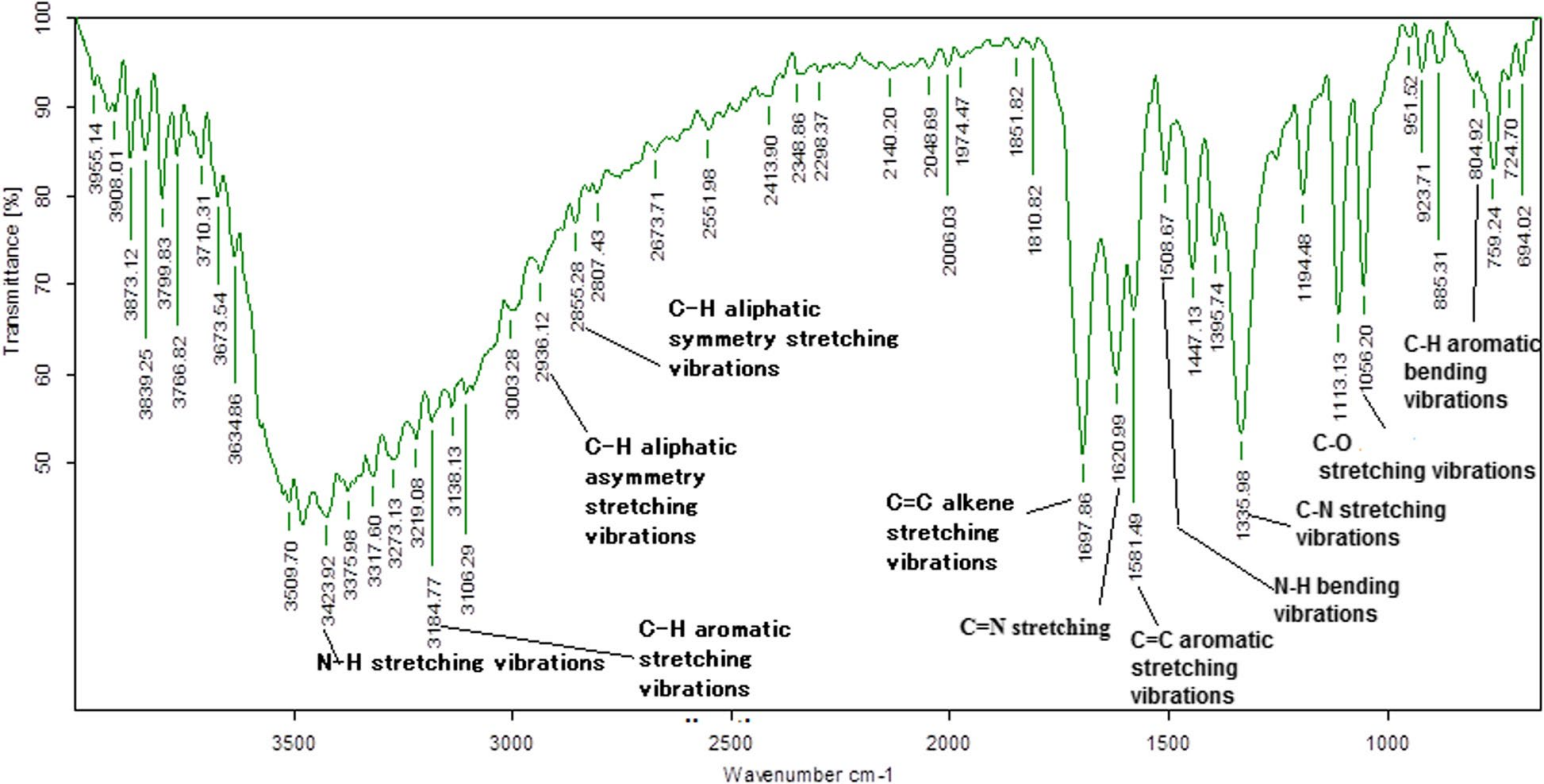
FTIR data showing various functional group peaks corresponding to the alkaloids

The sample was subjected to FT-IR analysis. The obtained FT-IR spectra (Fig. 11) revealed several regions correspond to characteristic IR absorption (cm^− 1^) of alkaloids such as N-H (3423), C-H (3184), C = C, C=O (1600, 1697), C = N (1620), C-N (1335), C-O-C (1056).

## Discussion

CRC claims millions of death every year in most developed countries. Now a day, it is the second most mortality-causing disease in worldwide. The most important risk factors are dietary, and genetic factors are mainly associated with CRC [38]. Recent statistics estimated that about one-third of colon cancer patients will be under the age of 50 years by the year 2030 [39]. Despite the huge improvements in the current treatment approaches, there is a significant increase in CRC cases. The mortality rate due to CRC is also associated with the serious side effects of current treatment [38].

Therefore, there is a necessity for identifying a novel, new, and specific anticancer agent for treating colon cancer, preferably with minimal side effects. Several reports suggested that due to the chemical diversity of natural products, specifically medicinal plants and their derived compounds always acts as a better source for novel anticancer drug discovery [40, 41]. Moreover, it is known that plant-derived compounds are associated with lesser toxicity than synthetic drugs. In addition, more than 50% of the current cancer chemotherapeutic agents were primarily obtained from plant sources [42, 43].

In the current study, we have demonstrated the anticancer effect of n-hexane extract of the root of *T. sinensis* (NHRT) against colon cancer (HCT-116 cells). Here, NHRT triggers ROS-induced apoptosis in the HCT-116 cells via PI3K/p-Akt/mTOR pathways. In general, reactive oxygen species (ROS) were frequently released during cellular metabolism. These species are highly reactive and have a very short span of life. Normally, the cancer cells like colon cancer require a high rate of ROS to maintain their physiological functions including growth and progression. However, forced upregulation of ROS beyond the threshold limit can induce oxidative stress and push the cancer cells toward death via activating various death pathways such as apoptosis. Whereas the normal cells in contrast to cancer usually maintain a strong adaptive mechanism and avoid cell death by preserving redox homeostasis.

Hence, medicinal plant extract or any plant-derived compounds that induce ROS can able to sensitize cancer cells to the treatment and can initiate cell death via activating multiple pathways [11, 44]. Here, we report that NHRT up-regulates the ROS, and induces apoptotic cell death. Cell death is mediated by cell cycle arrest, mitochondrial dysfunction, and mTOR inhibition [45].

Earlier, there are several compounds reported in other species of *Tinospora* such as tinosporide, furanolactone diterpene, furanolactone clerodane diterpene, furanoid diterpene, tinosporaside, tinosporine, magnoflorine, jatrorrhizine, palmatine, beberine, tembeterine, ß-sitosterol, and tinosporal acetate. Among these reported compounds, terpenoids, alkaloids, and steroids were known to possess promising anticancer potential. Specifically, jatrorrhizine, a proto-berberine alkaloid exhibits anticancer effects against colon cancer cells (HCT-116 and HT-29) through apoptosis via modulating Wnt/β-catenin signaling pathway [46]. Similarly, berberine isolated from other plant sources also showed an anticancer effect and induces apoptosis in breast and colon cancer cells. The anticancer effect of berberine is due to the alternation of JNK/p38/Wnt/catenin pathway, ROS upregulation, and mitochondrial dysfunction [47, 48]. Moreover, the GC-MS and FT-IR experiments revealed the presence of many alkaloids (Figs. 10 and 11). Furthermore, this data suggested that alkaloids such as berberine, jatrorrhizine, tembetarine, and palmatine either alone or in combinations among them, may be responsible for the observed anticancer activity of the NHRT.

Literature suggested that the mammalian target of rapamycin (mTOR) is always active in the HCT-116 cells. In cancer cells, mTOR is most important for protein synthesis via phosphorylating the essential molecules that regulate mRNA translation, and ribosome synthesis [49, 50]. Furthermore, mTOR lies the downstream of type I insulin-like growth factor receptor (IGHR). In response to ligand, IGFR gets activated and activates its downstream signals such as PI3K and Akt. The earlier studies suggest that the generation of any irregularity in the PI3K/Akt/mTOR pathway can lead to the development of cancer [51]. Hence, targeting mTOR, and mTOR- mediated signals like PI3K and Akt using plant-derived compounds can develop a promising therapeutic tool to fight cancer. Natural compounds such as curcumin, fisetin, indole-3-carbinol, epigallocatechin gallate, celastrol, quercetin, and resveratrol were reported to target mTOR-mediated pathways and reported to be can be useful in various cancers [51].

NHRT targets and modulate mTOR and its downstream signals and induces apoptotic cell death in HCT-116 cells through ROS generation and mitochondrial dysfunction. In addition, NHRT also modulates the expression of the caspase family, specifically Caspase-3, 7 & 9 in HCT-116 cells, which can enhance apoptosis in cancer cells.

## Conclusion

In this study, we conclude that NHRT induces cell death in HCT-116 cells via the generation of intracellular ROS, and downregulation of PI3K/Akt/mTOR signaling pathways. This report is unique of its kind and reveals the mechanistic study of *Tinospora sinensis* species. Hence, the findings may provide a way for further investigation on this plant for lead drug discovery for colon cancer in near future.

## Supplementary Information

**Additional file 1: Fig. S1.** Western blots.

## Data Availability

All data generated or analyzed during this study are included in this published article.

## Abbreviations

MTT: Methyl thiazoldiphenyltetrazolium bromide
GC-MS: Gas Chromatography Mass Spectrometry
FTIR: Fourier Transform Infrared Spectroscopy
Hela: Henrietta Lacks cervical cancer cells
MCF-7: Michigan cancer foundation-breast cancer
ROS: Reactive oxygen species
PI3K: Phosphoinositide 3-kinase
Akt: Protein Kinase B
mTOR: Mammalian target of rapamycin
NHRT: n-Hexane extract of *Tinospora sinensis* root
CRC: Colorectal cancer
DMEM: Dulbecco's Modified Eagle Medium
DCFDA: 2,7-dichlorofluorescein
DAPI: 4',6-diamidino-2-phenylindole
EATR: Ethyl acetate extract of *Tinospora sinensis*
ELTR: Ethanol extract of *Tinospora sinensis*
CON: Control
STD: Standard
