# Supplementary material for: Tinospora sinensis (Lour.) Merr alkaloid rich extract induces colon cancer cell death via ROS mediated, mTOR dependent apoptosis pathway: “an in-vitro study”
Source: BMC Complement Med Ther. 2023 Feb 3;23:33. doi: 10.1186/s12906-023-03849-5 (PMC9896699; doi:10.1186/s12906-023-03849-5)
Supplement: Supplementary file 1 — Additional file 1: Fig. S1. Western blots. [file 12906_2023_3849_MOESM1_ESM.pdf]

Western blots

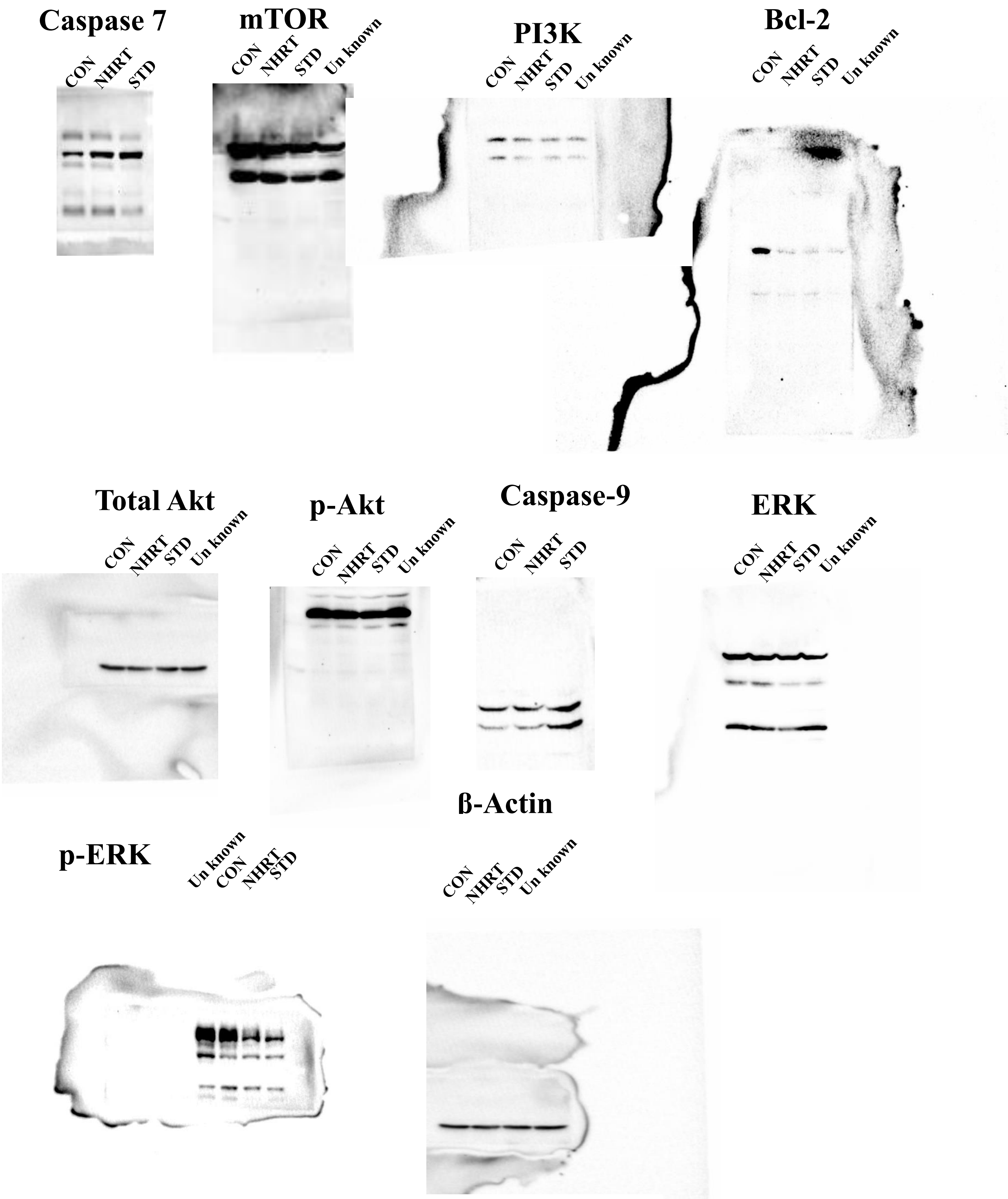

Figure S1: Western blots
